# A 4-Day Mindfulness-Based Cognitive Behavioral Intervention Program for CFS/ME. An Open Study, With 1-Year Follow-Up

**DOI:** 10.3389/fpsyt.2018.00720

**Published:** 2018-12-20

**Authors:** Bjarte Stubhaug, Haldis O. Lier, Jörg Aßmus, Arvid Rongve, Gerd Kvale

**Affiliations:** ^1^Department of Research and Innovation, Fonna Hospital Trust, Haugesund, Norway; ^2^Department of Clinical Medicine (K1), Faculty of Medicine and Dentistry, University of Bergen, Bergen, Norway; ^3^Centre for Clinical Research, Haukeland University Hospital, Bergen, Norway; ^4^Department of Mental Health, Haukeland University Hospital, Bergen, Norway; ^5^OCD-team, Haukeland University Hospital, Bergen, Norway

**Keywords:** chronic fatigue syndrome, myalgic encephalopathy, CFS/ME, stress reduction, cognitive behavioral therapy, acceptance and commitment therapy, 4-day concentrated treatment program

## Abstract

**Background:** Chronic Fatigue Syndrome/Myalgic Encephalopathy (CFS/ME) is an incapacitating illness in which single treatment interventions seem to have variable effects. Based on an earlier study we have conducted a new study with a concentrated intervention program. The aims of this study were to: (1) explore the clinical course for patients with CFS/ME who participated in a treatment program delivered during four consecutive days, and (2) evaluate their satisfaction with this program.

**Methods:** 305 patients diagnosed with CFS/ME (Oxford criteria), recruited from a clinical population referred to a specialist outpatient clinic, participated in an open uncontrolled study of the clinical course through 1 year. The study group participated in a 4-day group intervention program, comprised by education, cognitive group therapy sessions, mindfulness sessions, physical activity and writing sessions, within a context of cognitive behavioral therapy, mindfulness, acceptance and commitment model.

Assessments were done by self-reports prior to the first consultation, 1 week before and 1 week after the intervention program, and at 3 months and 1 year after the intervention. SPSS 23 and R 3.3 were used for statistical analyses. The associations between case definitions and the outcome measures (Chalder Fatigue Scale (FS), Short Form 36 (SF-36) physical functioning scale) were assessed by a linear mixed effects model (LME).

**Results:** Results showed statistically significant clinical changes for 80% of the patients after the intervention, changes being sustained through 1 year after the program. For both Fatigue Scale (FS) and the SF-36 there were statistically significant effects of time from baseline to all time points with a statistically significant drop in scores, applying the linear mixed effects model.

A subgroup fulfilling the inclusion criteria from the PACE study (Chalder Fatigue Scale >6/11, SF-36 Physical functioning <65/100) showed clinically significant improvement through 1 year, changes in outcome measures were statistically significant (*p* < 0.001). None of the patients included in the program dropped out, and a great majority of patients expressed high satisfaction with the content, focus and amount of treatment. **Conclusion:** Clinical changes observed from pre-treatment to 1 year follow-up could represent effects of the 4-day concentrated intervention program, and should be further explored in a controlled study.

## Background

Chronic Fatigue Syndrome (CFS), also named Myalgic Encephalopathy (ME), is an incapacitating illness characterized by severe and excessive fatigue, accompanied by a wide variety of health complaints involving several physiological organ systems including sleep problems, pain and cognitive dysfunctions, and with a general and often severe functional impairment (1, 2). The illness has recently been proposed redefined, to Systemic Exertion Intolerance Disease (SEID), emphasizing the functional impairment and core symptoms (3), although this definition as well as other case definitions of CFS/ME are being disputed (4, 5).

Despite substantial research effort, no intervention has yet been proven universally effective (6). The most promising treatment so far seem to be cognitive-behavioral treatment programs (7, 8) and graded exercise (7, 9, 10), although the effectiveness of interventions and robustness of findings are continuously being questioned (11). Effects are mainly on self-reported symptom reduction as well as improved quality of life and general functioning (2, 8, 12).

It has been suggested that the variability in treatment response might be related to differentiated needs from subgroups of CFS/ME patients (13). In line with this, we have previously examined the effects of a comprehensive treatment program where several treatment modalities were combined, e.g., educational sessions and body-awareness training sessions, as well as instructions of graded exercise (14), with promising effects.

Based on our earlier study (14), we have concentrated and intensified the program. In the current study, the treatment was delivered during four consecutive days. The program consisted of previously documented effective components: psychoeducation and counseling (15, 16), stress management (17, 18), mindfulness-based cognitive-behavior therapy/acceptance and commitment therapy (ACT) (8, 19–22), writing therapy (23), physical exercise (7, 9), mindfulness training (24–26), and acceptance (27, 28). Some of these elements have been combined in previous studies and intervention programs including mindfulness and acceptance, and have been increasingly applied in recent years and therefore require a thorough evaluation (18, 21, 29).

The aims of the study were (1) to study the clinical longitudinal course in chronic fatigue syndrome with two different case definitions, through changes from pre- to post-intervention including a 4-day concentrated treatment program, and (2) to study the patients' satisfaction with and acceptance of the content, format and effect of the intervention program.

## Methods

### Subjects

Three hundred five patients (84% women) with a mean age of 39 years (*SD* = 11.4) were recruited from a clinical population referred from general practitioners and specialist hospital clinics (neurology, infectious medicine, general medicine) to a specialist outpatient clinic for stress and psychological medicine in Western Norway, during 2009–2013. The patients participated in 45 different groups, each group with 8–10 patients, over a period of 45 months.

The therapy groups also included patients with non-CFS diagnoses, where the clinician found it relevant to offer this concentrated treatment approach.

The clinical study included patients who fulfilled inclusion criteria of CFS and who did not have any other medical illness giving reason for exclusion.

All patients who fulfilled inclusion criteria of the study and who were willing to participate in a group therapy program were offered participation in the study. All of these accepted. Twenty patients were not interested in participation because of the group format and, thus, were not offered participation in the program.

### Diagnostics

All participants fulfilled the Oxford criteria for chronic fatigue syndrome (30). These criteria require fatigue to be the main symptom, accompanied by significant disability in the absence of an exclusionary medical or psychiatric diagnosis, with a minimum duration of 6 months. Before inclusion in the study, patients went through a comprehensive medical examination. Relevant medical and laboratory tests performed recently prior to first consultation were accepted, and new or extended tests were performed when judged important in order to exclude any medical condition that could explain the fatigue complaints.

A semi-structured psychiatric interview, M.I.N.I. (31), was performed at the initial consultation by an experienced psychiatrist (BS), to examine any exclusionary psychiatric illness. The full range of comorbid conditions were not documented further.

All patients were also examined according to the CDC (1994) case definition of chronic fatigue syndrome (32). In accordance with CFS-CDC case definition, comorbid conditions of mild to moderate depression or anxiety were allowed. A subgroup of patients with CFS (CDC) was defined.

After referral, the patients received self-report questionnaires, which they returned at the first consultation (baseline).

### Measures and Instruments

Chalder Fatigue Scale (FS) (33) is a self-reporting scale which covers both mental and physical fatigue. The scale has acceptable psychometric qualities (34). FS consists of 11-items, each with four levels, which yield a range from 0 to 33 (35). The alternative bimodal scoring system (0 and 1 = 0 and 2 and 3 = 1), which yields a range from 0 to 11, was also used in the current study. According to this bimodal scoring system, a score of 4 or more is considered to indicate substantial fatigue (36).

Short Form Health Survey-36 (SF-36) is a validated self-rating questionnaire which measures health-related quality of life (HRQoL) (37). The SF-36 comprises 36 items that describe eight dimensions of functioning, where a higher score on the subscales represents better HRQoL. In the current study the dimension Physical functioning was employed. The validity of the Norwegian version of SF-36 has been found satisfactory (38). In the present study, changes in HRQoL were calculated by subtracting the follow-up SF-36 scores from the baseline SF-36 scores.

The Client Satisfaction Questionnaire-8 (CSQ-8) is an 8-item widely used self-report questionnaire which covers the patients' satisfaction with quality, content, amount as well as effects of the treatment. The scale has known and acceptable psychometric qualities (39). The questionnaire was administered 1 week after treatment. Items from the CSQ-8 include questions such as “Did you get the kind of service you wanted?” “Have the services you received helped you deal more effectively with your problems?” “If a friend were in need of similar help, would you recommend our program to her?” and “If you were to seek help again, would you come back to our program?” Items were rated on a Likert scale, ranging from 1 (quite dissatisfied/no, they made things worse/no, definitely not/poor/none of my needs were met) to 4 (very satisfied/yes, they helped a great deal/yes, definitely/excellent/almost all of my needs have been met).

The Beck Depression Inventory (BDI-II) assessed level of depressive symptoms. This is a 21-item self-report questionnaire which measures depressive symptoms during the 2 weeks prior to assessment (40). The BDI has well established psychometric properties. Higher total scores indicate more severe depressive symptoms; 0 to 13 represent “minimal” depression, scores from 14 to 19 are “mild,” scores from 20 to 28 are “moderate,” and scores from 29 to 63 are “severe” depression (41). The BDI scale has good internal consistency (42).

The Beck Anxiety Inventory (BAI) is a 21 item self-report questionnaire measuring the subjective, somatic, or panic-related symptoms of anxiety (43). Psychometric evaluations have reported adequate internal consistency and several studies have supported the reliability and validity of this instrument (44). The scoring of the BAI ranges from a normal level of anxiety (scores <7), mild anxiety (score range 8–15), moderate anxiety (score range 16–25) to severe anxiety (scores >26).

Internal consistency measured with Cronbach's alpha for the Chalder Fatigue Scale was 0.865, for SF-36 Physical Function 0.897, for BDI (depression score) 0.843 and for BAI (anxiety score) 0.878.

### Interventions

#### The Initial Consultation (T0)

Aims of the initial consultation were to assess the clinical condition, confirm the diagnosis of CFS/ME and to establish a positive therapeutic alliance. Patients were offered a medical explanation of their illness, based on a model of predisposing, precipitating, and perpetuating factors. Explanation of excessive fatigue and exertion intolerance was based on models of physiological activation, dysregulation and sensitization processes. Illness behavior was addressed and cautiously challenged, presenting a simple program trying to establish alternative strategies concerning sleep, physical activity and nutrition. Patients were also given a brief introduction to mindfulness exercises to practice daily (24, 45). They were encouraged to get up at approximately the same time every morning, restrict daytime sleep to 20 min, having regular meals through the day and short (30–60 min) daily walks at low pace. We also gave a brief information of the concentrated 4-day group program at this consultation.

The time lap between the initial consultation and the intervention program was 6 weeks (mean), but varied according to availability of the next program, as well as private matters concerning family, work or intercurrent illness.

#### The 4-Day Program

Prior to the groups, the patients were provided with an explanation of relevant maintaining factors, as well as basic principles for how to regulate the symptoms. Based on this information, they made a decision to allocate four consecutive days to the treatment.

One of the main features of this 4-day program is to deliver a composite of treatments that previously have been documented effective (7–9) or promising (14, 27), during four consecutive days. In order to allow the treatment to have full attention, we encouraged the patients to have no other appointments during these days. The 4-day format has, with a different content, been applied for OCD as well as for panic disorder, with highly promising results (46, 47) and low drop-out-rate.

#### Education

During the 4-day group program, the participants received two sessions of lecture /education (1st and 3rd day), each lasting 2 h. These sessions covered introduction to stress medicine with focus on physiological and psychological stress, sustained activation, dysautonomia, immune activation, and physiological dysregulation. The concept of sensitization (48, 49) was also presented, adressing some of the widespread subjective health complaints in CFS/ME (49).

The main illness model of CFS/ME in this program comprises both physiological and cognitive activation, sustained arousal and sensitization mechanisms, the effect of sleep disturbances and dysfunctional activity level, as well as focus on the illness behavior in CFS/ME and the frequent fear of exertion and further impairment.

In the educational sessions, patients were encouraged to ask questions for clarification, and alternative symptom explanations (e.g. energy loss vs. fatigue representing immune activation) were discussed and challenged or brought to the therapy groups.

A coping model with focus on stress expectation, positive outcome expectancy and active regulation of health complaints and symptoms was introduced (50). We tried to communicate clearly that regardless of causes, regulation of symptoms and change might be possible. However, a willingness to challenge existing illness perceptions and illness behavior is needed, as well as a commitment to test suggested coping strategies.

#### Mindfulness Sessions

The participants were introduced to guided mindfulness ad modum Kabat-Zinn (45, 51), and participated in mindfulness sessions twice daily (~30 min), in addition to several shorter sessions of 5–10 min integrated in the walking sessions.

#### Cognitive Group Therapy Sessions

The patients participated in group sessions 1–2 times daily, each lasting 30–60 min. In the very first session, each patient gave a brief outline of their illness history (5–10 min), without feedback from each other or from the therapist. After this, they were encouraged not to talk about illness and symptoms during breaks or leisure time, but to share experiences of success as well as plans for specific, observable changes they were going to make after the 4-day intervention, related to patterns of activity and rest.

During group therapy sessions the patients presented their concerns and typical coping strategies related to regulation of fatigue symptoms, e.g. sleep and physical activity. If not presented by the patients, topics like perfectionism and expectations from others, overachievement and fear of failure were introduced by the therapist. Acceptance and tolerance of subjective distress was included in the discussion, along with motivation for change and commitment by making decisions of change. Sessions were semi-structured, allowing for individual and group-specific dynamics, also allowing for topics introduced in educational sessions. Experiences from the mindfulness practices were frequently presented, aiming at strengthening the experience of self-efficacy and self-regulation.

#### Writing Experience

During 3 days of the group program, the patients were instructed to write for 15 min about positive experiences and emotions (23, 52). They were encouraged to select one or two experiences and elaborate upon these. This material was not examined by anyone else.

#### Physical Activity

The intervention program included daily walking sessions of 60–90 min, in low to moderate pace (aiming at HR <125). This activity included frequent stops and mindfulness sessions 5–15 min, focusing on being mindful of observing all senses at the present moment, encouraging the acceptance of disturbing thoughts and distressing physical sensations.

### Procedure

After the initial consultation and interview, lasting 2 h, all patients received written information regarding the group intervention program. The interval between the first consultation and the program was 1–46 weeks; the median time interval was 6 weeks, interquartile range (IQR) 7 weeks.

The group intervention program lasted for 4 days with 8 to 10 participants in each group. The participants stayed at a hotel nearby the clinic, in a rural area with an average travel distance of 2–3 h from home. The daily program lasted from 9 a.m. to 3–5 p.m. (A detailed description of the daily schedule is presented in Supplement Table 1).

All patients had a 60 min individual consultation with a therapist during the program, discussing individual issues of concern.

After the program, patients were encouraged to report by email or phone about their condition after approximately 1 month, but no obligations were made. One week, 3 months and 12 months after the intervention program the patients completed the self-report questionnaires again. The “Client satisfaction questionnaire” (CSQ) evaluating the program was reported 1 week after completion of the program.

### Subsample

All patients fulfilling Oxford criteria for Chronic Fatigue Syndrome comprised the main sample (*n* = 305). In order to allow for comparisons with other clinical studies, a sample consisting of patients with a bimodal score of 6/11 or more on the Fatigue Scale and a score of 65/100 or less on SF-36 “Physical functioning” subscale was also constructed (*n* = 148). The inclusion criteria for this subsample were chosen according to general clinical consensus of cut-offs for substantial fatigue, referring to inclusion criteria in the treatment effect study PACE (7).

This sub-sample used to compare results with PACE was divided into two groups; patients only meeting the Oxford criteria (*n* = 54) and patients fulfilling the CDC criteria (*n* = 94).

### Statistical Analysis

Descriptive methods were used to characterize the sample. The association between diagnoses (case definitions, Oxford and CFS-CDC) and the outcomes (fatigue scale (FS), SF-36 Physical Functioning) was assessed by a linear mixed effects model (LME) using case definitions, time and their interaction as predictors. We estimated the model adjusted for age, sex and the time between baseline (T0) and T1 one factor at the time as well as all together in a second model. For the final interpretation, we used the best of the models based on Akaike's Information Criterion (AIC) and the likelihood ratio test. Additionally we repeated the same procedure for the use of melatonin or anti-depressive medications as predictors instead of diagnosis (univariate models) as well as for all the predictors in the same model (multivariate model).

The Wilcoxon signed rank test was used to analyze change in Fatigue Scale, SF36 Physical functioning, BDI (depression) and BAI (anxiety) from baseline assessment to 1 year follow up (variables were not normally distributed). Effect sizes (*d*) were computed for the difference in mean scores for CFS and SF36 from pre- intake to follow- up assessments.

We compared the mean values of SF-36 Physical Functioning in this study sample to the population norm from Norway (38). The population norm data were adjusted by age and gender (53). We calculated effect sizes to compare the mean values of SF-36 Physical functioning subscale in this study population to the population norm, by subtracting the mean scores of the population norm from the mean score of the patient group divided by the standard deviation of the patient group. Effect sizes <0.2 are considered as trivial, from.2 to <0.5 as small, from.5 to <0.8 as moderate and >0.8 as large (54).

The significance level was set to 0.05. A Bonferroni adjustment for multiple testing was done in the main analysis (LME), setting the α level to.0083 (6 different models).

A goodness of fit evaluation for the linear mixed effects model was done, showing the model has acceptable fit for the data. The statistics for the goodness of fit evaluation are presented in Supplement Table 2.

The computation was done using SPSS 23 (IBM Corp. Armonk, NY) and R 3.3(55) with the package nlme 3.1(56) and the graphics was created by Matlab 2016a (The MathWorks Inc., Natick, MA).

### Ethics

All participants provided written informed consent prior to assessment. The study was approved by the Regional Committees for Medical and Health Research Ethics Committees (REC Western Norway) and the Norwegian Social Science Data Services (NSD). The study was performed in accordance with The Helsinki Declaration of the World Medical Association Assembly.

## Results

### Sociodemographic and Clinical Characteristics

Demographic and clinical characteristics for total population and subsamples are reported in Table 1. For the total study population, patients at pre-treatment assessment had a mean fatigue score (Fatigue Scale) score 24.8 (range 6–33, *SD* = 4.8). Ninety-seven percent of the patients had a score of 4/11 or more on the bimodal FS score, representing substantial fatigue. Mean score of SF-36 Physical functioning subscale was 60.9 (range 0–100, *SD* = 21.8). The effect size was large (effect size = 1.3) for the difference between this sample and the population norm regarding SF-36 Physical functioning. Gender and age adjusted population norm has a mean of 90.0. The mean depression (BDI-II) score was13.8 (range 0–53, *SD* = 7.5) representing minimal depression, the mean BAI (anxiety) score was 10.3 (range 0–52, *SD* = 7.9) representing mild anxiety.

**Table 1:** Sociodemographic characteristics in total population and diagnostic subgroups.

	**All**	**CFS-CDC**	**CFS-Oxford**
*N* (%)	305 (100)	171 (56)	134 (44)
Age (mean, SD, years)	39.3(11.4)	36.9 (11.8)	42.4 (10.2)
Gender female (n, %)	257 (84.3)	143 (84.6)	112 (83.6)
Education (n, %) <10yrs/10-13/>13yrs	28/129/140 9/43/47	18/62/72 11/37/43	10/51/68 8/40/52
Married/living in relationship/living alone (*n*, %)	147/52/69 48/17/23	77/32/43 45/19/25	70/20/26 52/15/19
Income in NOK (n, %) <300^*^/300-500/ >500	119/82/15 39/27/5	72/45/5 42/26/2	47/37/10 35/28/5
Work status (%) –employed –sick leave –sickness disability –support of family/student/unemployed/ other	35% 29% 26% 10%		
Beck depression inventory (mean, SD)	13.8 (7.4)	13.1 (6.8)	14.6(8.1)
*Beck anxiety* inventory (mean, SD)	13.8 (7.4)	13.1 (6.8)	14.6(8.1)
Melatonin	10.3 (7.9)	10.0(8.2)	10.7(7.6)
Anti-depressive medication	19 (6.3)	5 (2.9)	14 (10.5)

* *Income in thousand Norwegian Krones (NOK)*.

Two-hundred and 19 patients (72%) completed all assessments at pre-treatment, 1 week and 1 year follow-up. Patients completing all assessments had higher mean age than patients who did not complete all assessments [40.4 (*SD* = 11.4) vs. 35.5 (*SD* = 10.9), *p* = 0.006]. We found no statistically significant differences in pre-treatment scores of Fatigue Scale (FS), SF-36 Physical functioning, BAI, BDI or gender between completers (*n* = 219) and non-completers (*n* = 86) at 1-year follow-up. At one week follow-up 290/305 patients completed assessments, at 1 year follow-up 219.

### Change From Pre-treatment Assessment to Follow-Up 1 Week and 1 Year

The patient population showed statistically significant improvements on FS, SF-36 Physical functioning subscale, BDI and BAI from pre- treatment to 1-year follow up (all *p*-values were <0.001). For the primary outcome measures (FS and SF-36 Physical functioning subscale), effect sizes for the difference between pre-treatment and 1-year follow- up measure were large (>0.8). At 1-year follow up the patients had a mean BDI score of 8.0 (range 0–32, *SD* = 6.9) representing minimal depression, a mean BAI score of 6.4 (range 0–35, *SD* = 6.4) representing normal level of anxiety, and a mean score of SF-36 Physical functioning subscale of 77.4 (range 0–100, *SD* = 20.0). The effect size was moderate (effect size = 0.6) for the difference between this sample and the population norm regarding SF-36 Physical functioning. The patients had a mean FS score of 16.0 (range 0–33, *SD* = 6.6), 56% had a score of 4/11 or more on the bimodal FS score, representing substantial fatigue.

**Figure 1:**
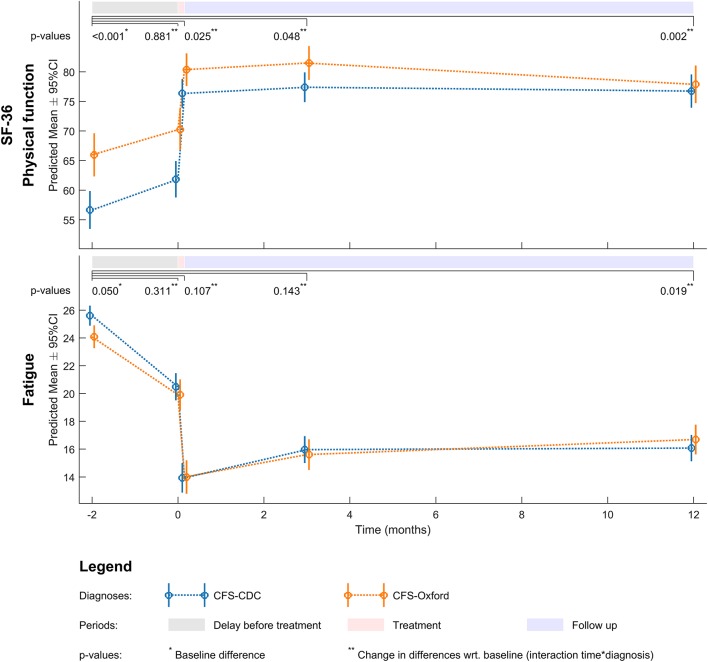
Changes in outcome measures by timeline and case definitions CFS.

For the fatigue scale (FS) there was a statistically significant effect of time from baseline to all time points with a statistically significant drop in scores, see Figure 1 and Table 2. The effect of case-definitions (Oxford /CDC) was statistically significant only at 1-year follow up with a greater reduction in the group with more severe symptoms at baseline (CDC). The use of melatonin did not affect the results and the use of anti-depressive medication had a small impact (3 points on FS scale 0–33) on the score after the group intervention. Regarding SF-36 there was a statistically significant effect of time to all time points, and a statistically significant effect of case definitions at the end of the intervention program and at 1 year follow-up, with no effect of melatonin and a small effect of anti-depressive medication at 1 year follow-up, see Figure 1 and Table 2.

**Table 2:** Improvements in symptoms from baseline to 1-year follow up (*n* = 305).

	**Pretreatment**		**One-year follow-up**		
	**Mean**	**SD**	**Mean**	**SD**	p^*^
Fatigue scale	24.8	4.8	16.0	6.6	<0.001
Fatigue scale, mental fatigue	8.1	2.2	5.4	2.9	<0.001
Fatigue scale, physical fatigue	16.9	3.3	10.5	4.6	<0.001
SF36, physical function	60.9	21.8	77.4	20.0	<0.001

* *Wilcoxon signed rank test*. *The global score also spans two dimensions—physical fatigue (measured by items 1–7) and mental fatigue (measured by items 8–11)*.

A clinically useful difference (7) between the means of the primary outcomes was defined as 0.5 of the SD of these measures at baseline (57) equating two points for FS and seven points for SF-36 Physical functioning. The proportions of patients who improved between baseline and 1-year follow up by two or more points of the CFS scale and seven or more on SF36 Physical functioning were calculated (Table 4), showing large increase in score in 80–90% of the participants.

Based on the clinical case inclusion criteria in the PACE study (7) (with a bimodal score of 6/11 or more on the Fatigue Scale and a score of 65/100 or less on SF-36 Physical functioning subscale), we constructed a subgroup of patients who fulfilled these criteria at baseline. We analyzed the change in outcome measures in this group, showing that a substantial proportion of the patients reported clinical improvement after 1 year, with mean change FS 26.7–17.0 = 9.7 (*p* < 0.001, d = 1.3); mean change SF-36 Physical functioning 44.1–70.3 = 26.2 (*p* < 0.001, d = 1.2), (Tables 3, 4).

**Table 3:** Subsample of patients ≥18 years, bimodal FQ≥6, and SF 36 physical function ≤ 65 (PACE criteria), *n* = 148.

	**Pretreatment**	**One year follow up**	**Comparison pretreatment and 1 year follow-up, *p, d***
Age	39.3 (11.3)		
Fatigue scale	26.7 (4.0)	17.0 (6.9)	< 0.001, *d =* 1.3
SF-36, Physical function	44.1 (14.5)	70.3 (22.7)	< 0.001, *d =* 1.2
BDI	14.8 (7.9)	8.2 (6.5)	< 0.001, *d =* 0.8
BAI	11.0 (8.3)	6.4 (5.9)	< 0.001, *d =* 0.6

**Table 4:** Comparison of change in Fatigue scale and SF-36 Physical functioning from pretreatment to 1 year after treatment in the two diagnostic subgroups.

	**Fatigue**			**SF-36 Physical functioning**		
	**Oxford**	**CFS- CDC**	Comparison, p^**^	**Oxford**	**CFS- CDC**	Comparison, p^**^
Pretreatment(t0)	25.7(4.4) (*n* = 54)	27.3(3.7) (*n* = 94)		46.7(14.0) (*n* = 54)	42.6(14.5) (*n* = 94)	
One year follow-up(t2)	18.4(6.1) (*n* = 46)	16.0(7.3) (*n* = 68)		67.7(21.1) (*n* = 46)	72.1(23.6) (*n* = 68)	
Change(t0-t2)	7.1(6.7)	10.8(7.4)	0.005	20.4(19.1)	29.2(23.4)	0.109
Number improved^*^ from baseline	84.8% (*N* = 39)	91.2% (*N* = 62)	0.371	78.3% (*N* = 36)	86.8% (*N* = 59)	0.306

* *Participants improved by two or more points for fatigue and seven or more for SF-36 Physical functioning*.

** *Mann-Whitney U-test*.

### Acceptance and Satisfaction With Treatment

**Figure 2:**
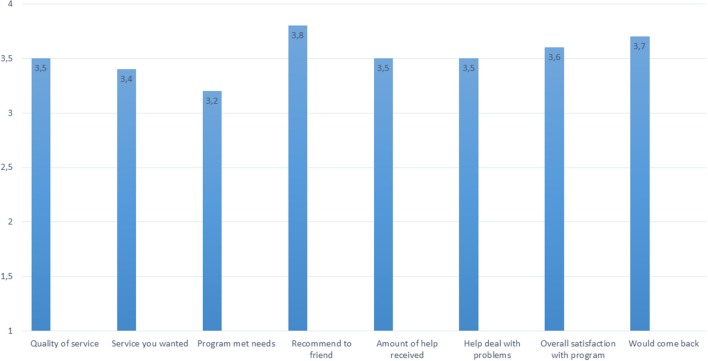
Acceptance and satisfaction with treatment program (CSQ-8). How would you rate the quality of service you have received?: 3.5 (SD = 0.6). Did you get the kind of service you wanted?: 3.4 (SD = 0.5). To what extent has our program met your needs?: 3.2 (SD = 0.7). If a friend were in need of similar help, would you recommend our program to him or her? 3.8 (SD = 0.4). How satisfied are you with the amount of help you have received?:3.5 (SD = 0.5). Have the services you received helped you to deal more effectively with your problems?: 3.5 (SD = 0.6). In an overall, general sense, how satisfied are you with the service you have received?:3.6 (SD = 0.5). If you were to seek help again, would you come back to our program? 3.7 (SD = 0.5).

None of the patients included (*n* = 305) dropped out of the 4-day program. The participants reported great satisfaction with treatment by CQS-8 questionnaire (range 1–4), see Figure 2. The single item “overall, general satisfaction” had a mean score of 3.6 (*SD* = 0.5).

## Discussion

The results from this open study indicate that a brief, concentrated treatment program for CFS/ME might be highly beneficial. Patients reported improvement in outcome measures representing both clinical and statistical significance. This is interesting, as most studies on effectiveness in CFS/ME interventions generally have shown low or modest effect. Most participants in this study had been impaired by fatigue and ill health for a long time without improvement, indicating that time in itself is insufficient.

The number of participants (305), the statistical significance of improvements and the persistent changes through the 1-year follow up also indicate an effect of the program, more than what is expected by time or usual medical care.

The study population probably represents a selection bias with a positive attitude of the treatment approach before intervention, and this might have influenced the reported outcome. This is, however, a situation in clinical real life, where the profile of a clinic and the reputation of the therapists are known or accessible for the patients, and may influence the acceptance of treatment as well as the active participation and outcome. It seems reasonable to assume that this also is an important factor for the unusually low dropout during the program. As we examined the motivation for participating prior to inclusion, not inviting the patients who explicitly refused the program, this probably contributed to a low dropout as well.

Many patients with CFS/ME tend to be critical to biopsychosocial interventions, and possibly most of these patients did not accept referral to the clinic, contributing to the possible selection bias. Then again, the patients included in the study did all fulfill criteria for Chronic Fatigue Syndrome, and considering the large number of participants, they clearly represent a CFS population, albeit not representing the total body of CFS/ME patients.

The intervention program was motivated by earlier clinical work and research studies (49, 58), with an aim of establishing a more concentrated, time-effective, and clinical effective intervention program. The therapeutic rationale behind the program was to increase the medical knowledge and interpretation of bodily distress, challenge and modify dysfunctional illness perceptions as well as illness behavior, and through acceptance and commitment strategies contribute to behavioral change and clinical improvement.

By and large, this was recognized and accepted by the patients in this study. One week after the intervention the participants expressed high satisfaction with the 4-day program in terms of content, quality as well as the amount of help they received. More importantly, they regarded the program as helpful with respect to their problems. The patients' initial evaluation was supported by self-reported highly significant change. Furthermore, the self-reported improvement reported 1 year after the intervention was large, also compared to previous studies.

Examining a subgroup defined as functionally impaired, using the criteria for clinical improvement defined in the PACE study (7) (Fatigue Scale and. SF-36 Physical functioning), the proportion of patients reporting significant improvement 1 year after treatment was nearly 90% (Table 4).

It is interesting to note that the clinical status and changes reported through 1-year follow-up course in the Oxford and the CFS-CDC case definition groups are quite identical, but that the most severely impaired group at baseline (CDC) reported the greatest improvement by 1 year. This result differs from our earlier RCT study (14), where we found the group with the greatest impairment at baseline to have the least improvement after intervention. The actual intervention is a more concentrated format (4 days) in combination with a more comprehensive content of the program, focussing on restoring sleep, emphasizing more the acceptance of the present status, and challenging more actively the illness perceptions of CFS/ME. Possibly, such a concentrated format allowing for extensive education and challenge of cognitive illness perceptions and behavioral patterns of avoidance, as well as offering an existential frame of acceptance of the present situation could be a key effectiveness factor. This is also in accordance with similar concentrated intervention formats (47).

At 1 year follow-up, half of the patients completing assessments (56%) still report levels of fatigue representing substantial fatigue (> 4/11 Fatigue Scale). Whether this reflects characteristics of the sample, such as heterogeneity and severity, or is related to inadequacies of the treatment interventions (59), is not possible to decide based on the current study design.

In this study, patients were offered additional medical treatment for comorbid conditions, especially for sleep disturbance and depression. When controlling for the possible effects of melatonin and antidepressants, the clinical changes from pre- to post intervention and through 1-year follow-up were not influenced by melatonin medication, while use of antidepressants from baseline to 1 week pre-treatment had a weak influence on changes within this period. Since antidepressants were prescribed based on clinical judgment of a comorbid depression, medication might have had an effect also on fatigue symptoms. Nevertheless, the impact of medication seems overall insignificant.

The feasibility of doing such a concentrated intervention program in regular clinical practice should be good, as the current program was actually carried out in a clinical practice, part of the national health care system. The low drop-out rate also indicate that such a program is feasible as well as acceptable for a clinical population.

The current study has obvious methodological limitations. The open study design does not allow for comparison with control groups, making conclusion of the actual effect of the treatment program and its impact on the clinical course through the follow-up period difficult.

Whether the reported improvement and symptom reduction represent the effect of the program or merely represent the time effect, return to homeostasis or regression to the mean, is not possible to determine due to the study design and available data. Possibly, a selection bias from a majority of patients having a positive attitude to the clinic and the treatment approach might also influence the outcome. The outcome measures based on self-report represent a methodological challenge, as self-reported improvement and objective functional improvement may differ (60). Nevertheless, this is the general method of outcome measures in clinical studies on CFS/ME. There was only one therapist conducting the intervention program through the study period. This represents a strength in terms of a consistent approach across the intervention period, while it also makes it difficult to generalize whether the treatment approach is robust and feasible in clinical setting with several therapists with different training.

Still, the results are promising, with respect to the acceptance, satisfaction as well as acute and long-term clinical improvements. The number of patients included is substantial, and even though the design does not allow for identification of specific factors that might have contributed to the results, the highly promising results might be a starting point for a controlled study.

## Conclusion

A brief, concentrated intervention program is well tolerated by patients with CFS/ME, and clinical changes reported from pre-treatment to 1-year follow-up are substantial with high satisfaction reported by patients.

## Ethics Statement

This study was carried out in accordance with the recommendations of ethical guidelines by REK Norway and with written informed consent from all subjects. All subjects gave written informed consent in accordance with the Declaration of Helsinki. The protocol was approved by the REK Vest (Regional Ethics committee), Norway.

## Author Contributions

BS designed the study, conducted the clinical work, and wrote the first draft of all parts of the manuscript. GK participated in the design of the study and in the writing of all parts of the manuscript. HL participated in all parts of the manuscript and participated in the statistical analyses. JA conducted the LME statistical analyses. AR added medications to the database and critically reviewed the manuscript.

### Conflict of Interest Statement

The authors declare that the research was conducted in the absence of any commercial or financial relationships that could be construed as a potential conflict of interest.
