# Supplementary material for: A 4-Day Mindfulness-Based Cognitive Behavioral Intervention Program for CFS/ME. An Open Study, With 1-Year Follow-Up
Source: Front Psychiatry. 2018 Dec 20;9:720. doi: 10.3389/fpsyt.2018.00720 (PMC6306445; doi:10.3389/fpsyt.2018.00720)
Supplement: Supplementary file 1 [file Table_1.pdf]

Supplement: Table 1

Description of daily schedule in 4-day concentrated program

Day 0:
Patients arrive in the evening (some have several hours travel distances), for dinner and one hour presentation of the program and practical issues.

Day 1:
(Through the program, there are breaks of 15 minutes between 45 minutes sessions, where people can rest, sleep, talk, drink coffee/tea or take a short walk.)

09 am-10: introduction of the program, presentation of participants (names, short social status).
10-11: Lecture/presentation of basic concepts of stress medicine, autonomic nervous system, stress and restitution (sympathetic and parasympathetic systems), physical and mental/ cognitive symptoms arising from overactivation and sustained arousal: physiological dysregulation, dysautonomia, immune activation, negative emotional responses, sleep disturbances.
11-12: short presentation of individual illness histories, with a clear instruction not to include traumatic or serious emotional events, focusing on precipitating factors and actual symptoms and impairment.
(The rationale for this instruction is to avoid emotional arousal from other patients' histories, to keep focus on being here with their present status of health, recognizing their own symptoms and impairment with a feeling of coherence with the group, without disturbing emotional reactions).
12-1 pm: lunch
1pm-1.30: writing exercises, exploring thoughts and feelings from one of the most positive memories/events they can recall at this moment.
1.30-2.30pm: continuing educational session (lecture and questions/ discussion): presenting concepts of vulnerability, various causes of activation (infection, disease, stress) and perpetuating factors in sustaining symptoms and impairment.
Introducing coping as a concept, both external/ instrumental coping and internal/ emotional coping.
Making clear this program is a coping-oriented program, where evidence-based knowledge might strengthen recognition and acceptance of the present state, including emotional reactions and dysfunctional illness behavior. Acceptance of and exposure to uncomfortable physical and emotional reactions are presented as conditions for change and improvement.
Through this and later sessions, the discussion of CFS/ME diagnosis is intentionally limited, giving room for this discussion later in the program.
2.30-3pm: mindfulness session
3-4pm: Hot meal
4-5.30 pm: Walk outdoor in the nature in the area (along the sea, in the woods, in the mountains). (Patients who feel incapacitated to participate are free to move lightly at individual pace with simple mindfulness instructions.)
Walking at slow pace with low/moderate heart rate (<120), many short stops and one long session (20 minutes) with mindfulness exercises, focusing on senses: body, breath, landscape, light, sounds, smells, touch of wind and sun. Recognizing and accepting

physical discomfort and thoughts being anywhere but here… bringing the attention and awareness back to the present moment of being here….
5.30 pm: end of program, free time. Patients are encouraged not to go on the internet or social media or having unnecessary phone calls (except family), but rather rest, take a walk, write and do mindfulness exercises.
Most patients have supper at the same time/ same table, and some stay there for most of the evening. Speaking of their illness history, impairments or negative emotions are clearly discouraged (which is accepted and approved by the vast majority of patients).

Day 2:
09-10: Mindfulness exercise.
10-11: Cognitive-oriented group session, exploring physical and mental stress responses, challenging common illness coping strategies, e.g. excessive rest or sleep.
11-12: group therapy session, topics of illness behavior and acceptance
12-1pm: lunch/ rest/ writing therapy session (15 minutes)
1-2 pm: group session, focus on possible sources of support and motivation
2-3pm: mindfulness session
3-4pm: Hot meal
4-5.30pm: Walk outdoor with integrated mindfulness sessions

Day 3:
09-10: mindfulness exercise
10-11: educational session, expanding on stress medicine, acceptance, exposure to greater activity with possible post exertional malaise, need for physiological and emotional regulation.
11-12: walk outdoor with mindfulness exercise
12-1pm: lunch, rest/ writing therapy (15 minutes)
1-2pm: educational session, emphasizing further and continuous work with acquired illness coping strategies.
2-3pm: group session, focus on exposure and regulation
3 pm: end of group program, individual consultation (1hr) with therapist

Day 4:
09-10: mindfulness exercise
10-12: presentation of individual goals and plans for committed actions in terms of change in some daily routine/ behavior/activity. Challenging the actual commitment in this.
12-1pm: lunch and evaluation
1-2.30 pm: motivational session with focus on positive outcome expectancy and adherence to the illness coping program.
2.30 pm: end of program, return to home.
